# Fluid shear stress and endothelial cells synergistically promote osteogenesis of mesenchymal stem cells via integrin β1-FAK-ERK1/2 pathway

**DOI:** 10.3906/biy-2104-20

**Published:** 2021-12-14

**Authors:** Mingli JIANG, Qihua SHEN, Yi ZHOU, Wenxia REN, Miaomiao CHAI, Yan ZHOU, Wen-Song TAN

**Affiliations:** 1 State Key Laboratory of Bioreactor Engineering, East China University of Science and Technology, Shanghai China

**Keywords:** Mesenchymal stem cells, endothelial cells, coculture, fluid shear stress, osteogenesis, integrin β1

## Abstract

Prevascularization and mechanical stimulation have been reported as effective methods for the construction of functional bone tissue. However, their combined effects on osteogenic differentiation and its mechanism remain to be explored. Here, the effects of fluid shear stress (FSS) on osteogenic differentiation of rat bone-marrow-derived mesenchymal stem cells (BMSCs) when cocultured with human umbilical vein endothelial cells (HUVECs) were investigated, and underlying signaling mechanisms were further explored. FSS stimulation for 1–4 h/day increased alkaline phosphatase (ALP) activity and calcium deposition in coculture systems and promoted the proliferation of cocultured cells. FSS stimulation for 2 h/day was selected as the optimized protocol according to osteogenesis in the coculture. In this situation, the mRNA levels of *ALP*, runt-related transcriptional factor 2 (*Runx2*) and osteocalcin (*OCN*), and protein levels of OCN and osteopontin (OPN) in BMSCs were upregulated. Furthermore, FSS and coculture with HUVECs synergistically increased integrin β1 expression in BMSCs and further activated focal adhesion kinases (FAKs) and downstream extracellular signal-related kinase (ERK), leading to the enhancement of Runx2 expression. Blocking the phosphorylation of FAK abrogated FSS-induced ERK phosphorylation and inhibited osteogenesis of cocultured BMSCs. These results revealed that FSS and coculture with HUVECs synergistically promotes the osteogenesis of BMSCs, which was mediated by the integrin β1-FAK-ERK signaling pathway.

## 1. Introduction

One of the current limitations in bone tissue engineering is the inability to provide sufficient blood supply in the inception phase after transplantation, leading to cell death in engineered tissue constructs. Preconstruction of a vascular network through coculture of osteogenic cells (osteoblasts or mesenchymal stem cells (MSCs)) and vasculogenic cells (endothelial progenitor cells or endothelial cells (ECs)) is one of the crucial methods for accelerating the fusion with the host vasculature and increasing the survival and regeneration of bone tissue (Kang et al., 2014; Kocherova et al., 2019). The direct coculture of MSCs and ECs has been found to promote osteogenic differentiation and the formation of a prevascular network in vitro (Heo, et al., 2019). In addition to vascularization, bone vascular systems are exposed to environments with mechanical loading in vivo (Gusmão et al., 2009). Lack of mechanical stimulation leads to osteoporosis, bone calcium loss, and bone loss (Zuo et al., 2015). Therefore, it is essential to apply mechanical stimulation on the coculture system to construct bone microtissue.

Fluid shear stress (FSS) on the surface of bone cells is caused by the flow of interstitial fluid driven by mechanical loading and bending of bones, which generates biochemical signals in bone cells, thereby exerting biological effects (Wittkowske et al., 2016). Studies have shown that FSS has significant effects on MSCs function; in particular, it regulates the proliferation and expression of osteogenic markers (Li et al., 2004; Elashry et al., 2019). Corrigan et al., (2018) demonstrated that applying FSS to MSCs significantly promoted the early osteogenesis of MSCs and increased the expression of osteogenic genes *Cox2* and *OPN*. Furthermore, the magnitude, frequency, and duration of shear stress are correlated with cell behaviors such as gene expression and mineralization of MSCs (Stavenschi et al., 2017). Since mechanical stimulation and coculture with ECs are both crucial for bone formation and remodeling, their combined effect on bone may have a more significant therapeutic effect. However, most studies focus on osteogenesis in monocultured MSCs, whereas the combined effects and potential mechanism of FSS applied to MSCs-ECs cocultured system remain poorly understood.

Cell sense FSS through mechanoreceptors, such as integrins and ion channels, thereby driving a dynamic cascade of intracellular signals to regulate cell differentiation (Huang et al., 2018). Integrins are a superfamily of more than 20 alpha/beta transmembrane heterodimers, which connect the extracellular matrix and cytoskeleton (Takada et al., 2007). Integrins are mechanosensitive, and it was previously verified that integrin α_V_β3 and integrin β1 can respond rapidly to FSS in less than 1 min (Li et al., 2005). Upon activation of integrins, signals can be transmitted to the nucleus by altering the conformation of the cytoskeleton (Wang et al., 2009) or by activating integrin-mediated focal adhesions including focal adhesion kinases (FAKs) or Src signaling (Thompson et al., 2013). FSS-induced integrins can significantly improve osteogenesis. Liu et al., (2014) found that FSS stimulation on hMSCs increased ALP activity and expression of osteogenic gene expression through the integrin β1-ERK1/2 pathway in a perfusion culture system. Moreover, it has been found that 3D-printed MSCs-human umbilical vein ECs (HUVECs) coculture upregulate integrins from HUVECs in the growth medium (Piard et al., 2019). However, the role of integrins involved in FSS and coculture combined system is unclear.

The study aimed to investigate the effects of FSS on osteogenesis in bone marrow-derived stem cells (BMSCs) based on coculture with HUVECs to explore the underlying mechanism. The effects of FSS on cell morphology and proliferation were investigated, and the mechanical conditions for osteogenic differentiation in the coculture system were optimized. FSS was demonstrated to promote osteogenic differentiation of BMSCs in the coculture system through the integrin β1-FAK-ERK1/2 signaling pathway. These results indicate that mechanical stimulation in combination with a MSCs-ECs coculture system is an effective method for engineering prevascularized bone tissue. 

## 2. Materials and methods

### 2.1. Cell isolation and culture

Sprague Dawley rats (SPF grade, four-week-old, male) of 80 g~120 g were bought from Shanghai SLAC Laboratory Animal Co., Ltd. The rats were sacrificed by cervical dislocation, and the bones were isolated aseptically. BMSCs were isolated by bone marrow adherence method (Jin et al., 2018) and cultured in α-MEM (Gibco, USA) containing 10% fetal bovine serum (FBS; Hyclone, USA) at 37 °C with 5% CO_2_. Cells at passage 3 to 5 were used. Antibodies used for characterization of BMSCs and the results are shown in Supplementary Table 1 and Supplementary Figure 1. 

HUVECs were purchased from ScienCell and cultured in endothelial cell growth medium (ECM; ScienCell, USA) supplement with 8% FBS and 100× endothelial cell growth supplement (ECGS). Cells at passage 3 to 8 were used in our experiments.

### 2.2. Induction of osteogenesis

In all experiments, BMSCs were seeded at a density of 0.5×10^4^ cells/cm^2^ in 6-well plates. In the coculture system, BMSCs and HUVECs were cocultured in direct contact at a 1:2 ratio in growth medium (α-MEM with 10% FBS and ECM at a 1:1 ratio). After one day of cell adhesion, the growth medium was replaced with osteogenic induction medium (OIM) composing of Dulbecco’s modified eagle medium (Gibco) supplemented with 10% FBS, 10^−7^ M dexamethasone (Sigma, USA), 10 mM β-glycerol phosphate (Sigma), and 50 μg/mL L-ascorbic acid (Sigma). ECGS (100×) were used to maintain ECs survival in the coculture system. Monocultured BMSCs were cultured in OIM. In p-FAK inhibitor test, 5 μM PF-573228 (PF; Beyotime, China) dissolved in DMSO was added to OIM. 

### 2.3. Application of FSS to cultured cells

Cells were incubated in a rocking culture system (DLAB, China) and subjected to FSS cycles. We used a fixed rocking angle of 7^o^, frequency of 60 rpm, and fluid depth of 2.08 mm. These parameters ensure that the cells were always covered with medium during the mechanical rocking cycle. Assuming a fluid viscosity of 10^–3^ Pa·s, the FSS at the bottom center of a 6-well plate is 37.5 mPa (0.375 dyn/cm²) according to the FSS formula reported previously (Zhou et al., 2010). The description of the rocking culture system and the calculation of FSS are provided in Supplementary Figure 2. 

### 2.4. Staining of the cytoskeleton

After two days of FSS stimulation in the proliferation medium, cells were fixed with 4% paraformaldehyde in PBS for 15 min, and then permeabilized with 0.1% Triton X-100 for 10 min. After rinsing three times with PBS, cells were stained with phalloidin (50 μg/mL) labeled with TRITC (Solarbio, China) and 6-diamidino-2-phenylindole (DAPI, Solarbio) for 40 min and rinsed three times in PBS. Cells were observed by inverted fluorescence microscope (Nikon Eclipse Ti-S, Japan).

### 2.5. DNA content determination

After rinsing with PBS, cells in 6-well plates were treated with 800 μL of 0.1% papain solution containing 2.5 U/mL papain (Sigma), 5 mM EDTA, 5 mM L‐cysteine, and 0.1 M Na_2_HPO_4_ (pH 6.2) and treated at 60 °C overnight. Lysate was collected into a 1.5 mL centrifuge tube and centrifugated at 4000 rpm for 5 min. Then, 50 μL of supernatant was added to 2 mL of Hoechst 33258 working solution (pH 7.4) containing 100 ng/mL in TNE buffer, 50 mM Tris-HCl, 100 mM NaCl, and 0.1 mM EDTA. The fluorescence intensity at 350 nm was measured using a fluorometer (Hoefer DQ300, USA). 

### 2.6. Osteogenic differentiation assay

ALP activity was detected after 7 days of osteogenic induction. The cells were rinsed with PBS and fixed with 4% paraformaldehyde in PBS for 15 min. And 800 μL BCIP/NBT solution (Beyotime) was added to 6-well plates for 1 h. The chromogenic reaction reflects ALP activity, which was quantified by alkaline phosphatase assay kit (Nanjing Jiancheng Biological Engineering Institute, China) following the manufacturer’s instruction.

Calcium deposition was detected after osteogenic induction for 14 days. The cells were rinsed with PBS and fixed with 4% paraformaldehyde in PBS for 15 min. After rinsing with PBS, cells in 6-well plates were stained with 1% Alizarin red S (Sigma) for 30 min. The chromogenic reaction reflects calcium deposition. Calcium content was quantified by calcium detection kit (Nanjing Jiancheng Biological Engineering Institute) following the manufacturer’s instruction.

### 2.7. Quantitative real-time PCR (RT-PCR)

Total RNA was extracted from cells using Trizol reagent (Invitrogen, USA) and 1 μg RNA was used for cDNA synthesis using MLV reverse transcriptase (Promega, USA) according to manufacturer’s instruction. Then quantitative real-time PCR was performed in a 20 μL reaction system, using SYBR mix (Roche, Switzerland) following the manufacturer’s instruction. PCR conditions were as follows: 95 °C for 10 min followed by 40 cycles of amplification at 95 °C for 10 s, 60 °C for 20 s, and 72 °C for 30 s. GAPDH was used as a housekeeping gene. Gene expression was analyzed by ΔΔCT method. Primers sequences are listed in Supplement Table 2.

### 2.8. Western blot

Proteins from cells were obtained in protein extraction reagent RIPA lysis buffer (Beyotime) with 10 mM phenylmethylsulphonyl fluoride (Beyotime) at 4 °C for 30 min. The supernatant was harvested after centrifugation at 12000 rpm for 30 min. A BCA assay kit (Beyotime) was used to determine protein concentration. Then, equal amounts of protein samples were added to the loading buffer (Beyotime). After heating at 95 °C for 5 min, each sample was run on a 10% SDS polyacrylamide gel. The separated proteins were electrically transferred onto PVDF membrane (Millipore,USA), which were then blocked with 5% (w/v) no-fat dry milk in TBST for 1 h at room temperature. After blocking, the membranes were incubated with primary antibodies (Supplement Table 3) overnight at 4 °C, followed by incubation in horseradish peroxidase (HRP)-conjugated goat anti-rabbit (Abcam, UK) for 1 h. Immunoreactive bands were visualized by enhanced chemiluminescence (Millipore) and quantitative analyzed by Image J software. β-actin was used as an internal control standard to analyze relative protein expression.

**Table:** Supplementary Table 3. Primary antibodies used for Western blot.

Antibody	Dilution	Source	Code number
β-actin	1:1000	Cell Signaling Technology	TA-09
OCN	1:1000	Abcam	Ab14173
OPN	1:1000	Abcam	Ab8448
Integrin β1	1:1000	Cell Signaling Technology	#4760s
FAK	1:1000	Cell Signaling Technology	#3285T
Phospho-FAK	1:1000	Absin	Abs 131023
ERK1/2	1:1000	Cell Signaling Technology	#4695
Phospho-ERK1/2	1:1000	Cell Signaling Technology	#4377
Runx2	1:1000	Cell Signaling Technology	#12556

### 2.9. Magnetic cell sorting

Cocultured cells were suspended by trypsin digestion, rinsed with PBS twice, centrifuged at 1500 rpm for 5 min, and collected in 1.5 mL centrifuge tubes. After rinsing with PBE buffer (PBS with 0.5% BSA and 2 mM EDTA, pH 7.2), each sample was retained 100 μL liquid, followed by incubation in CD31 antibody-labeled magnetic bead suspension (Miltenyi, Germany) at 4 °C for 30 min in a dark room. The samples were centrifuged at 1000 rpm for 5 min and rinsed with PBE buffer twice. Then 500 μL PBE was added buffer to obtain the cell suspension. The separation column (Miltenyi) was placed in a magnetic field and wet with 500 μL PBE buffer. Then, cell suspension was added to the separation column and eluted with PBE buffer, and negative cells (BMSCs) were collected. After microscopy of eluent containing 1–2 cells, the separation column was removed from the magnetic field, and PBE buffer was added to elute the positive cells (HUVECs). Cells were incubated with antibody anti-CD31 (PE-conjugated mouse IgG1, R&D, USA) according to the manufacturer’s instructions and analyzed on a flow cytometer (BD FACS Calibur, USA). 

### 2.10. Statistical analysis

All experiments were repeated for three times, and the experimental data were presented as mean ± standard deviation (SD). Statistical significance was determined using a two-tailed student’s t test. p < 0.05 was considered as a statistically significant difference.

## 3. Results

3.1. Eﬀects of FSS on cell morphology

**Figure 1:**
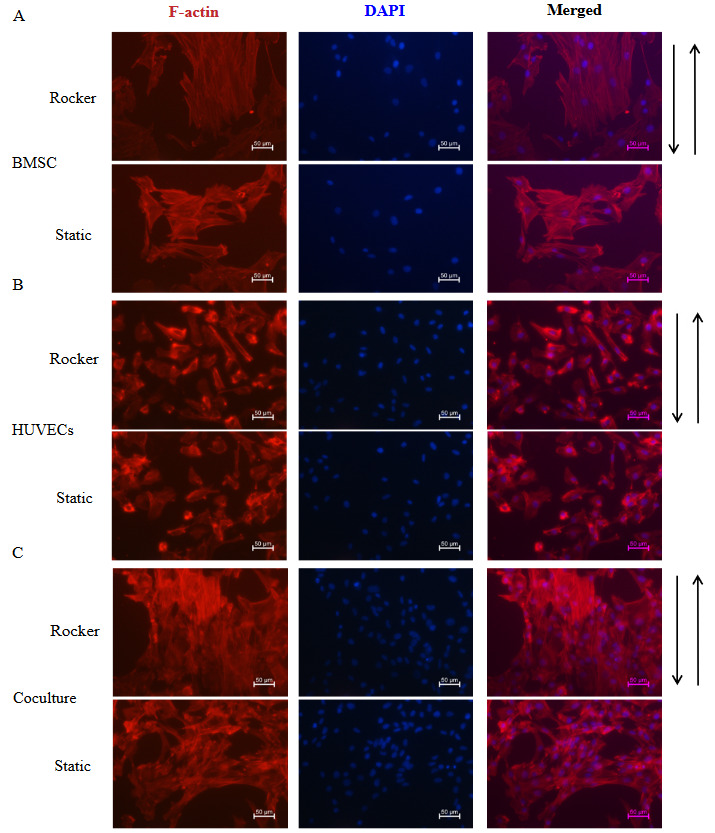
Staining of cytoskeleton. (A-C) The cells were stained with phalloidin to visualize F-actin (red) and DAPI (blue) to visualize the nucleus under FSS stimulation or static condition. Scale bars, 50 μm.

FSS (60 rpm, 1 h/day) was applied for 2 days, and the staining of cytoskeleton was performed to detect cell morphology. As shown in Figure 1A, the cytoskeleton of monocultured BMSCs loaded with FSS was mostly arranged along the direction of FSS compared with that of the static group. The cytoskeleton in the static HUVECs and the FSS-loaded HUVECs showed random orientation, only partial HUVECs were elongated under dynamic conditions (Figure 1B). Most of the cytoskeletal fibers in cocultured cells were arranged in the direction of the FSS stimulation compared with the static cocultured cells (Figure 1C). These results suggested that FSS stimulation-induced directed rearrangement of the cytoskeleton of BMSCs and cocultured group, thus affecting cell function.

### 3.2. Optimization of FSS application conditions of the BMSCs-HUVECs coculture system

**Figure 2:**
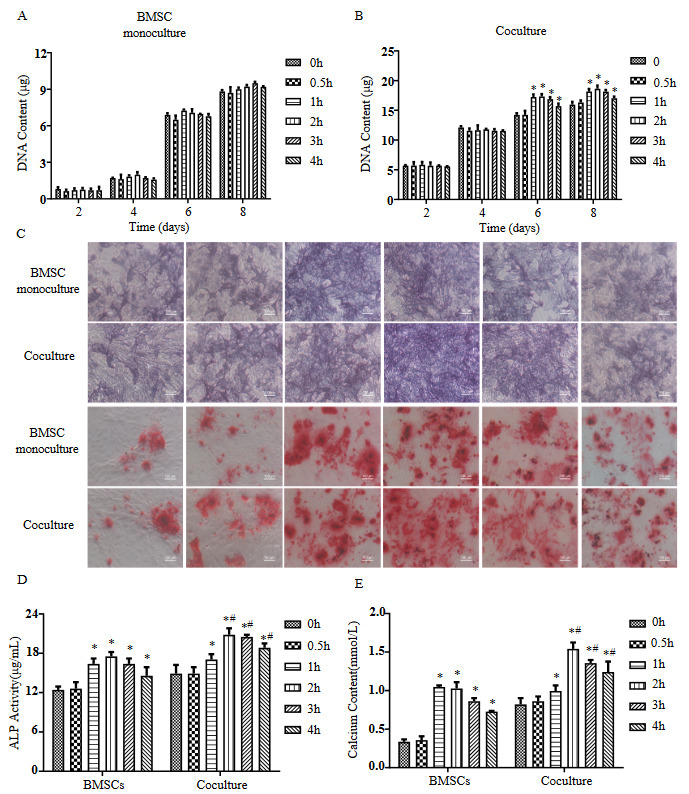
Proliferation and osteogenic differentiation of monoculture and coculture loaded with various FSS stimulus durations. (A and B) DNA content of monoculture and coculture. (C) BCIP/NBT staining on day 7 and Alizarin red S staining on day 14 in monoculture and coculture (scale bars, 100 μm). (D) Quantification of ALPase activity on day 7. (E) Quantification of calcium deposition on day 14. Compared with static group, *p < 0.05; compared with monocultured BMSCs, # p < 0.05. n = 3.

To investigate the effects of FSS on cell proliferation and osteogenesis, monocultured BMSCs and coculture cells were exposed to different durations of FSS (0 h, 0.5, 1, 2, 3, and 4 h per day). For the monocultured BMSCs, no significant difference in DNA content was observed between the static groups and FSS stimulation groups after 7 days (Figure 2A). For the coculture systems, treatment with FSS for 1, 2, 3, and 4 h per day all enhanced the DNA content on days 5 and 7 compared to that of the static coculture groups, and the DNA content decreased after FSS stimulation for 4 h (Figure 2B). The osteogenic differentiation assay revealed that groups with FSS stimulation for 1, 2, and 3 h had the strongest NBT/BCIP staining and the highest ALP activity, and those with FSS stimulation for 1 and 2 h had the strongest Alizarin Red staining and the highest calcium content in monocultured BMSCs (Figure 2C-E). For the coculture groups, FSS-loaded groups had the highest ALP activity at 2 and 3 h/day and the strongest calcium deposition at 2 h/day. When the stimulation period was prolonged to more than 2 h/day, ALP activity and calcium deposition content in the coculture group gradually decreased. Moreover, the ALP activity and calcium content in the coculture groups with FSS stimulation for 2, 3, and 4 h/day were significantly higher than those in the corresponding dynamic monoculture BMSCs. Hence, 2 h/day FSS stimulation was selected as the optimal loading conditions for the subsequent experiments.

### 3.3. Synergistic effects of FSS and coculture with HUVECs on osteogenic markers of BMSCs

**Figure 3:**
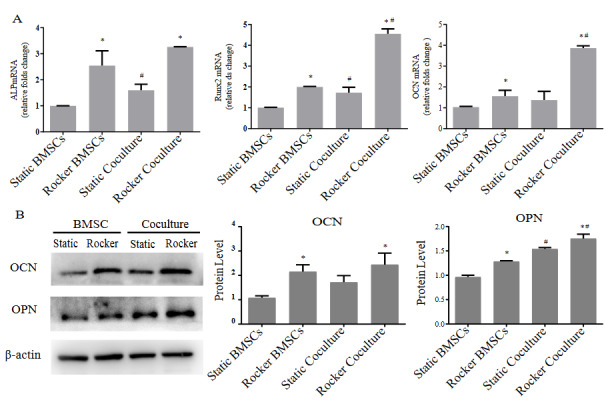
Osteogenic gene expression and osteogenic marker protein expression of monoculture and coculture with or without 2h/day FSS stimulation. (A) Expression of osteogenesis-related genes on day 7. (B) Expression of osteogenesis-related proteins on day 7. Compared with static group, * p < 0.05; compared with monocultured BMSCs, # p < 0.05. n = 3.

To further investigate the effects of 2 h/day FSS stimulation on osteogenesis of monoculture and cocultured cells, Cells were harvested on day 7 after 2h/day FSS stimulation, and the expression of osteogenic marker genes and marker proteins was investigated. Under static conditions, the *ALP* mRNA and *Runx2* mRNA of the coculture were significantly higher than those in the monoculture (Figure 3A). Compared with the respective static control group, *ALP*, *Runx2* and *OCN* expression significantly improved in the coculture and monoculture groups under FSS stimulation. Moreover, with the same levels of FSS, *Runx2* and *OCN* gene expression levels in the coculture groups were higher than those in the monoculture groups. The osteogenic marker proteins OCN and OPN in the monoculture and the coculture were upregulated and compared with those in the corresponding static control groups (Figure 3B). Furthermore, under static conditions and FSS stimulation, the coculture groups had higher OPN protein expression than the corresponding monoculture group. Overall, these results indicated that FSS stimulation for 2 h/day and coculture with HUVECs synergistically promote osteogenesis-related genes and proteins in BMSCs.

### 3.4. FSS and coculture with HUVECs together affect the expression of Integrin β1

**Figure 4:**
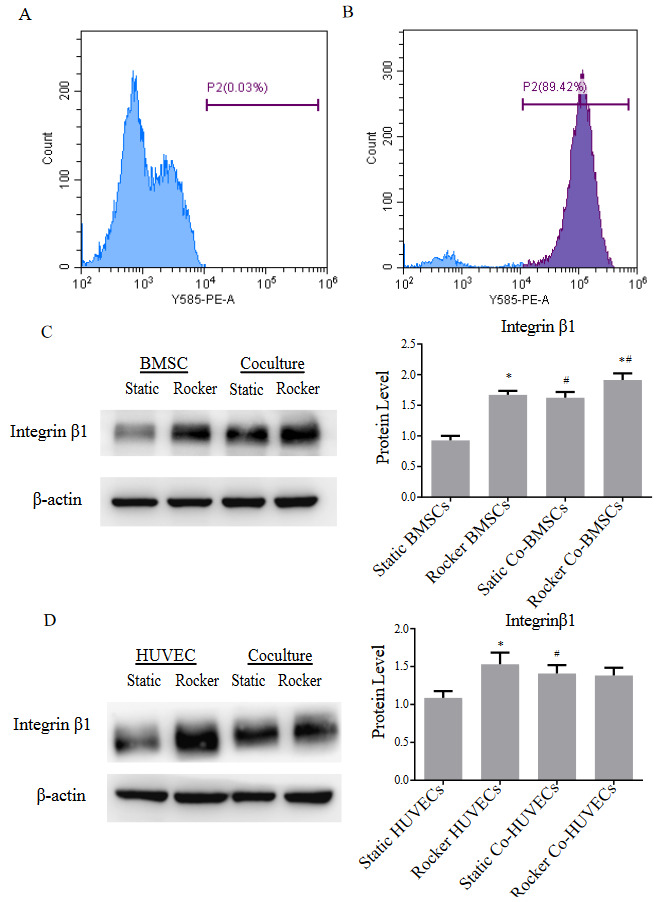
Cell sorting efficiency and integrin β1 expression of monoculture and coculture under dynamic and static conditions. (A-B) Detection of cell surface antigen CD31 of negative and positive cells after cell sorting. (C) Protein expression of integrin β1 in BMSCs. (D) Protein expression of integrin β1 in HUVECs. Compared with static group, * p < 0.05; compared with monocultured group, # p < 0.05. n = 3.

HUVECs were positively selected for the cell surface antigen CD31 by MACS, and BMSCs were harvested as negative cells after coculture for 1 day to distinguish the protein expressed by the two types of cells. According to the flow cytometry results of CD31, the negative cells contained only 0.03% CD31^+^ cells, and HUVECs reached 89.24% of positive cells after MACS sorting (Figure 4A, 4B). 

Integrins are known as mechanical signal receptors and can transduce mechanical signals into intracellular signals. Under static conditions, the protein levels of integrin β1 in BMSCs and HUVECs in the coculture were significantly higher than those in the corresponding monoculture group (Figure 4C, 4D). In addition, compared with static conditions, FSS promoted the integrin β1 expression in BMSCs in monoculture and coculture. Furthermore, the integrin β1 in rocker cocultured BMSCs showed a 17% increase compared to dynamic monoculture and a 19% increase compared to static coculture. These results indicated that FSS stimulation and coculturing synergistically promote integrin β1 expression in BMSCs. For HUVECs, although FSS stimulation can increase integrin β1 expression under monoculture conditions, there was no significant difference in integrin β1 levels between dynamic coculture and static coculture. 

### 3.5. FAK-ERK1/2 pathway was involved in FSS-enhanced osteogenesis of cocultured BMSCs

**Figure 5:**
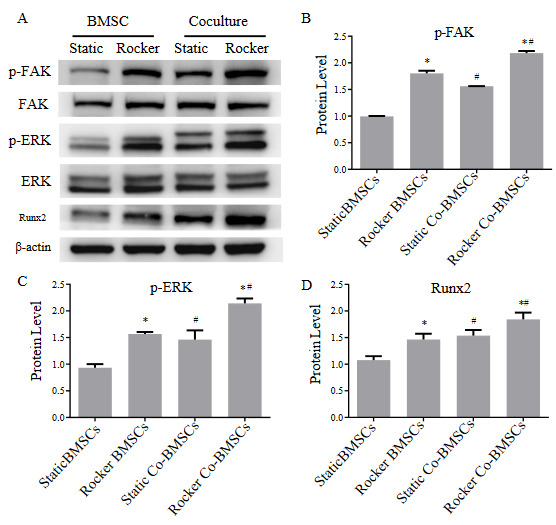
Effects of FSS stimulation and coculture on FAK-ERK-Runx2 signaling pathway in BMSCs. (A) The protein levels of p-FAK, FAK, p-ERK1/2, ERK1/2 and Runx2 of monocultured and cocultured BMSCs under 2h/day FSS application or not after 1 day. (B-D) Quantification of protein levels by Image J software. Compared with static group, *p < 0.05; compared with monoculture group, #p < 0.05. n = 3.

Studies have shown that activation of integrins can activate downstream FAK, ultimately affecting osteogenic differentiation. Under static conditions, the coculture groups had higher phosphorylation levels of FAK (Figure 5A and 5B), ERK1/2 (Figure 5A and 5C) and higher Runx2 expression (Figure 5A and 5D). Under FSS stimulation, the phosphorylation levels of FAK, ERK1/2 and Runx2 expression in the monoculture groups and the coculture groups were higher than the corresponding static culture group (Figure 5). The protein levels of FAK phosphorylation, ERK1/2 phosphorylation and Runx2 in dynamic coculture groups were all enhanced compared to dynamic monoculture group and static coculture group. Furthermore, these results showed p-FAK in BMSCs has the same expressive tendency as Integrin β1.

### 3.6. PF treatment inhibited the effect of FSS induced osteogenesis of cocultured BMSCs

**Figure 6:**
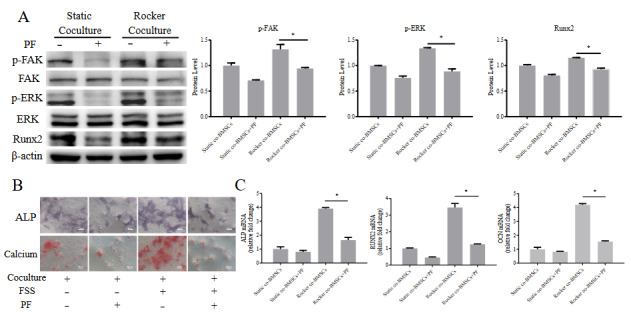
Effects of p-FAK inhibitor PF treatment on cocultured BMSCs. (A) The protein levels of FAK-ERK-Runx2 signaling pathway with/without PF under FSS application or not after 1 day. (B) NBT/BCIP staining on day 7 and Alizarin red S staining on day 14 with/ without PF under FSS application or not (scale bars, 100 μm). (C) Measurement of the osteogenic genes on day 7 with/without PF under FSS application or not.compared with static co-BMSCs group, * : p < 0.05; compared with Rocker co-BMSCs treated with PF group, #p < 0.05. n = 3.

To further validate that the FAK-ERK1/2 signaling pathway is an important mediator in FSS-induced osteogenesis of cocultured BMSCs, PF was added to OIM for inhibiting FAK phosphorylation. Following PF administration, p-FAK and p-ERK1/2 levels of cocultured BMSCs were down-regulated, the Runx2 protein level exhibited a 20% decrease compared to rocker cocultured BMSCs (Figure 6A). Furthermore, PF significantly reduced FSS-induced ALP activity on day 7 and calcium deposition on day 14 (Figure 6B), suppressed the increase of osteogenic related genes of *ALP*, *Runx2*, and *OCN* induced by FSS stimulation (Figure 6C). Totally, it was identified that integrin β1-FAK-ERK1/2 pathway was involved in the synergistic effects of osteogenesis of cocultured BMSCs under FSS.

## 4. Discussion

This study investigated the effects of FSS stimulation on osteogenesis of BMSCs cocultured with HUVECs. We found that FSS stimulation altered the cytoskeleton of monocultured BMSCs and cocultured cells. Moreover, intermittent application of FSS for various durations (1, 2, 3, and 4 h/day) promoted ALP activity and calcium deposition in BMSCs based on coculture with HUVECs, and FSS application for 2 h/day had the strongest ability to upregulate osteogenesis in coculture. Furthermore, we particularly found that HUVECs coculture and FSS synergistically upregulate integrin β1 in BMSCs, which activates the phosphorylation of downstream mediators FAK and ERK1/2, thereby upregulating Runx2 transcription to promote osteogenesis.

The cross-talk between osteogenic cells and vasculogenic cells influences the functions of cocultured cells. Fan et al., focused on a static coculture found that miR-200b is transferred from BMSCs to HUVECs through gap junctions to promote osteogenesis in BMSCs (Fan et al., 2018). In addition to coculturing, increasing evidence has confirmed that mechanical loading upregulates BMSCs proliferation and osteogenesis (Li et al., 2013; Stavenschi et al., 2017). Thus, HUVECs coculture combined with mechanical loading is a possible efficient method for bone tissue engineering. Jiang et al. applied 6% cyclic tensile strain on a BMSCs/VECs coculture system and finally found synergetically promotion of osteogenesis in BMSCs (Jiang et al., 2016). However, how BMSCs respond to FSS stimulation in BMSCs-HUVECs coculture and detailed molecular mechanism remain to be further elucidated.

To investigate this, we exposed a coculture system of BMSCs and HUVECs to FSS on a rocking see-saw system that generated FSS. The effect of FSS on the osteogenic differentiation of MSCs is related to the duration of stimulation. Kreke et al. found that MSCs loaded with 1.6 dyn/cm^2^ FSS for 30 min/day had the highest levels of *BSP* and *OPN* compared to 5 min/day and 120 min/day (Kreke et al., 2005). Lim et al. (2013) found that proliferation and ALP activity of MSCs tended to increase after 10 and 60 min/day of stimulation. To optimize the duration for osteogenesis of our coculture, we investigated the osteogenesis under different durations of FSS (0, 0.5, 1, 2, 3, and 4 h/day). The results indicated that FSS significantly promote osteogenesis of monocultured BMSCs, and FSS for 1 h/day resulted in the highest ALP activity and calcium deposition. For coculture groups, optimal osteogenesis was observed at 2 h/day, and the *ALP*, *Runx2* and *OCN* genes levels and OPN protein level were higher than those in static coculture and dynamic monoculture groups. These results suggested that coculture with HUVECs and FSS stimulation synergistically influence osteogenesis in BMSCs. However, a study has shown that FSS decreases the ALP activity and calcium deposition in flow perfusion coculture of hMSCs and HUVECs at 1:1 ratio (Dahlin et al., 2014), which is inconsistent with our results. In addition, Barron (2012) co-seed of MC3T3-1 and ECs at 49:1 ratio in perfusion showed higher osteogenic activity. On the one hand, the osteogenic characteristics of cells from different sources are different, on the other hand, the cellular interactions are affected by culture mode, such as 2D/3D culture, cell ratio, and FSS parameters. Thus, more experiments are needed to optimize and summarize the FSS application conditions.

Our results also revealed the enhancement of cocultured cell proliferation under FSS stimulation for 1-4 h/day compared with the static coculture, which was not observed in monocultured BMSCs. Considering FSS may change the interaction between two cells in coculture to regulate proliferation and osteogenesis, Luo et al. (2011) found that FSS of 1 dyn/cm^2^ has no significant effect on the proliferation of MSCs, while higher FSS inhibited the proliferation of MSCs. Another study showed that MSCs-loaded 3D scaffolds cultured under shear stress exhibit higher DNA content (Salgado et al., 2020). These phenomena are also closely related to the MSCs culture environment. In addition, we did not distinguish the contribution of BMSCs and HUVECs to proliferation in coculture; further investigation is needed. Although the duration of FSS has been optimized in our study, the physiological level of FSS is 8-30 dyn/cm^2^ (Weinbaum et al., 1994). Therefore, to simulate physiological FSS, we can focus on investigating the effect of another magnitude of FSS on coculture in future research.

Both BMSCs and HUVECs are mechanosensitive cells, and mechanical stimulation regulates cell functions by adjusting cell-cell crosstalk in coculture. Jiang et al. discovered that 6% cycle tension directly stimulate BMSCs, promoting the secretion of VEGF in BMSCs, which, in turn stimulates VECs to secrete soluble factors such as BMP-2 to promote osteogenesis in BMSCs (Jiang et al., 2018). In addition to soluble factors, integrins especially integrin β1, play a central role in mechanotransduction. In this study, FSS increased the integrin β1 levels in monocultured BMSCs and monocultured HUVECs. Litzenberger et al., (2010) demonstrated that inhibiting integrin β1 in MLO-Y4 cells abrogated the upregulation of Cox-2 mRNA and PGE2 release in response to oscillatory fluid flow. Moreover, we found that integrin β1 levels in BMSCs and HUVECs were both upregulated in coculture. TGF-β has shown significant effects on the expression of integrin β1 (Kumar et al., 1995; Chen et al., 2018), and it was proved that FSS regulated TGF-β1 secretion in VECs (Negishi et al., 2001). Meanwhile, the concentration of TGF-β in the coculture system was significantly increased in BMSCs-HUVECs coculture, which was mainly secreted by HUVECs (Fan et al., 2018). Thus, TGF-β1 may mediate cross-talk in coculture and further regulate the expression of integrin β1 under FSS, which remains to be further explored. In addition, FSS-induced cocultured BMSCs, but not cocultured HUVECs, had a higher integrin β1 expression than rocker the monoculture group and the static coculture group. All in all, these results suggested that FSS and coculture have synergic effects on osteogenesis through promoting expression of Integrin β1 in BMSCs.

Integrins are able to transmit signals by altering the conformation of the cytoskeleton (Wang et al., 2009). Previous studies have shown that loading with FSS adjusts rearrangement of MC3T3-E1 osteoblasts (Malone et al., 2007), and endothelial cells (Mengistu et al., 2011). In this study, FSS stimulation caused the cytoskeleton of BMSCs and coculture to rearrange in the direction of stress. It was proved that mechanical stimulation can induce cytoskeletal remodeling to enhance the nuclear translocation of YAP, which further promote osteogenesis of human periodontal ligament cells (Yang et al., 2018). However, whether FSS-induced cytoskeletal rearrangement mediates cocultured osteogenic differentiation in coculture remains to be explored. Moreover, we found that ECs showed a random orientation of cytoskeletal fibers under FSS stimulation. The average shear stress of ECs in the large veins, small veins, and vena cava of the human body is 0.524 Pa, 1.080Pa, and 0.300Pa, respectively (James et al., 2018). However, the peak value of FSS in our experiment was 37.5 mPa, which may not reach the threshold for the cytoskeleton rearrangement of ECs. Thus, we need to increase FSS magnitude in our coculture system to approach the physiological FSS.

It is known that through docking proteins, such as paxillin and tensin, β1 integrin can lead to the autophosphorylation of FAK (Schlaepfer et al., 1999). FAK has been reported to regulate the commitment of MSCs into the osteogenic or adipogenic lineages (Schreiber et al., 2019). In our study, p-FAK of cocultured BMSCs was upregulated synchronously with integrin β1 under FSS, treatment with PF inhibiting p-FAK abrogated FSS-induced osteogenesis and osteogenic marks gene expression in the coculture. FAK can activate downstream signal ERK1/2 and further promote Runx2 expression, thereby regulating osteogenesis (Kanno et al., 2007). Consistently, the p-ERK1/2 level and Runx2 level increased in coculture loaded with FSS, and inhibition phosphorylation of FAK reduced ERK1/2 phosphorylation and Runx2 expression. Hence, these findings indicated that FSS and coculture together activate ERK1/2 in BMSCs through integrin β1-FAK signaling, thereby upregulating the protein expression of Runx2 to promote the osteogenic differentiation.

In conclusion, our research revealed that FSS stimulation and coculture synergistically enhance the expression of integrin β1 in BMSCs, further increasing the expression of Runx2 through the Integrin β1-FAK-ERK1/2 signaling pathway to mediate osteogenic differentiation. These results preliminarily explore the mechanism of osteogenic differentiation in FSS-loaded coculture and provide a theoretical basis for FSS in vitro to regulate osteogenic differentiation of the BMSCs-HUVECs coculture system.

## Compliance with Ethical Standards 

All procedures performed on rats were in compliance with the guidelines of the ethics committee at East China University of Science and Technology.
